# Do Livestock Injure and Kill Koalas? Insights from Wildlife Hospital and Rescue Group Admissions and an Online Survey of Livestock–Koala Conflicts

**DOI:** 10.3390/ani11092684

**Published:** 2021-09-13

**Authors:** Alex Jiang, Andrew Tribe, Clive J. C. Phillips, Peter J. Murray

**Affiliations:** 1 School of Veterinary Science, The University of Queensland, Gatton 4343, Australia; zijian.jiang@uqconnect.edu.au; 2 Turner Family Foundation, Hidden Vale Wildlife Centre, 617 Grandchester Mount Mort Rd., Grandchester 4340, Australia; andrew.tribe@turnerfamilyfoundation.com.au; 3 Curtin University Sustainability Policy (CUSP) Institute, Curtin University, Perth 6845, Australia; clive.phillips@curtin.edu.au; 4 School of Sciences, University of Southern Queensland, Darling Heights 4350, Australia

**Keywords:** *Phascolarctos cinereus*, koala, cattle, cows, livestock, trauma, injury, attack, trample, domestic, wildlife conservation

## Abstract

**Simple Summary:**

Koala populations in Australia are declining and the species is vulnerable to extinction. In the past decade, grazing livestock emerged anecdotally as a threat to koala survival; cattle and horses were reported to have trampled koalas to death when encountered on the ground. In this study, we investigated the scale, frequency, and outcome of livestock-inflicted incidents to koalas via an online survey, and analysed koala admission records from Queensland wildlife hospitals and a wildlife rescue group (Wildlife Victoria) in Victoria. The results provide evidence of both livestock-inflicted injuries and deaths to koalas, especially as these have been confirmed by witness statements. The outcomes for the koala victims of the incidents were severe, which had a 75% death rate. The reported frequency of livestock–koala incidents was low but increasing, with 72 cases (0.14% out of 50,873 admissions) in Queensland wildlife hospitals during 1997–2019, and 59 cases (0.8% of 7017 rescue records) in Wildlife Victoria during 2007–2019, but it is recognised that this was likely to be under-reported. Future research is encouraged to explore the causes of livestock–koala incidents and to develop management strategies to minimise the livestock threat to koalas.

**Abstract:**

Koala populations in Australia are declining due to threats such as chlamydiosis, wild dog predation and vehicle collision. In the last decade, grazing livestock emerged anecdotally as a threat to koala survival in areas where koala habitat and livestock grazing land overlap. This is the first study investigating the significance of livestock-inflicted injuries and deaths in koala populations over a large spatial and temporal scale. We investigated the outcome, scale, and frequency of livestock–koala incidents via an online survey and analysed koala admission records in Queensland wildlife hospitals and a wildlife rescue group (Wildlife Victoria) in Victoria. The results provide evidence of both livestock-inflicted injuries and deaths to koalas, especially as these have been confirmed by witness statements. The outcomes for the koala victims of the incidents were severe with a 75% mortality rate. The reported frequency of livestock–koala incidents was low but increasing, with 72 cases (0.14% out of 50,873 admissions) in Queensland wildlife hospitals during 1997–2019, and 59 cases (0.8% of 7017 rescue records) in Wildlife Victoria during 2007–2019. These incidents were likely to be under-reported due to the remoteness of the incident location, possible mis-diagnoses by veterinarians and the possible reluctance of farmers to report them. Future research is encouraged to explore the mechanics and causes of livestock–koala incidents and to develop management strategies to minimise the livestock threat to koalas.

## 1. Introduction

As an iconic, cryptic species in Australia, koalas (*Phascolarctos cinereus*) live in eucalypt forests. However, over recent decades their populations have declined drastically [1]—in some populations by as much as about 80% [2,3]. The reasons include the historical hunting and pelt trade [4], habitat loss and fragmentation [5,6], disease [7], wild and domestic dog predation [8], collisions with vehicles [9], climate change [10] and bush fires [11].

In line with these major threats to the survival of wild koalas, a new threat has emerged recently—livestock. Anecdotal incidents of koala injuries or deaths caused by livestock have been reported in news media reports, social media and wildlife hospital records over the past decade. Based on a Google search, the first evidence was video clips, one showing cattle chasing a koala in a paddock [12], and another showing koala being chased from tree to tree by cattle exhibiting aggressive behaviour, including lowered heads, pawing the ground and warning vocalisations [13]. The first media report about the threat of livestock to koalas [14] appeared soon after a mother koala was witnessed being trampled to death by cattle in Victoria, which left the joey orphaned, and alleged incidents of wildlife killed by cattle were apparently known by farmers and wildlife carers. Later more livestock-inflicted deaths to koalas were reported in northern New South Wales, with at least eight koalas killed by livestock over 15 months during 2016 and 2017 [15], and in the Darling Downs region of Queensland “dozens of koalas” have been reported to be injured by cattle and horses every year [15,16].

Aside from the anecdotal media reports, there is a scarcity of published research exploring the significance of koala mortality and injury caused by livestock. In the first publication directly investigating livestock-related incidents involving koalas [17], admission records to Currumbin Wildlife Hospital in south-east Queensland from 2010–2016 were analysed, and showed 10 reports of koalas found in livestock paddocks (0.55% of total 1818 admissions). Five koalas were confirmed victims of cattle attacks, three had injuries suspected to have been caused by cattle, but not witnessed, and two were without trauma injuries [17]. These five out of eight injured koalas were euthanised or died in hospital, and three were successfully treated and released. Another study, based on koala admission records from four wildlife hospitals of south-east Queensland, indicated that five (0.2% of total admissions) of 2031 injuries, i.e., bone fractures in koalas, were caused by livestock trampling them in the period 1997 to 2010 [18]. Given the spatial and temporal limitations of these reports, it is likely that this problem is more widespread and under-reported because of the large overlap between livestock grazing land and koala habitat [19], especially in the coastal regions of Queensland, New South Wales, Victoria, and South Australia. Many koala ecologists (e.g., S. Fitzgibbon and J. Hanger 2018, pers. comm., 19 August), wildlife veterinarians (e.g., A. Gillett 2018, pers. comm., 19 August) and wildlife carers (e.g., C. Gover 2018, pers. comm., 20 April) believe that livestock, especially cows with their large size and potentially aggressive maternal drive [20], are an important threat for koala population viability within the grazing areas that koalas inhabit.

Given the sampling limitations of the only two relevant previous studies, i.e., Hill et al. [17], who only studied six years data from one hospital, and Henning et al. [18], who only focused on bone fracture cases, further research and investigation of the significance of livestock–koala conflict is required. This study aimed to investigate, or at least provide evidence for or against, the existence of livestock–koala incidents, and estimate the scale, frequency and outcome of reported livestock-inflicted injuries and deaths to koalas via an online survey, and analyses of records of koala admission to wildlife hospitals and a rescue group with a larger temporal and spatial scale across eastern Australia.

## 2. Materials and Methods

### 2.1. Online Survey

To estimate the extent of livestock–koala incidents, an online survey of livestock-inflicted injuries/deaths to koalas was conducted using the Checkbox™ survey platform from November 2019 to June 2020. The target recipients were: (a) veterinarians and nurses in wildlife hospitals and clinics; (b) koala carers in wildlife rescue groups; (c) wildlife/koala ecology researchers and conservation staff; and (d) livestock farmers. We sent survey invitations via emails and social media to various organisations and industry associations of a variety of target recipient groups. The contact information was sourced from Google and social media, using queries including but not limited to “wildlife hospital/clinic”, “wildlife rescue”, “wildlife researcher/ecologist” and “farmer/grazier/livestock/cattle/beef/dairy association”. We requested the recipients to circulate the survey invitation to their members or people of interest, and thus the total number of recipients was unknown. The survey can be found in Appendix A and a list of invitation recipients in Appendix B.

### 2.2. Admission Records

Due to difficulties in obtaining access to data and/or limited numbers of relevant records in some wildlife hospitals, and a lack of digitalised records in wildlife rescue groups, it proved difficult to source records of koalas injured or killed by livestock. However, we obtained access to two major datasets of koala admission records relevant to this study. One was from the Queensland Koala Hospital database (KoalaBase [21]), managed by the Queensland Government, Department of Environment and Science (DES). The database included admission records for koalas (including sightings and DOA (Dead On Arrival) cases from hospitals, clinics and rescue groups between 1997 and 2019, especially from the four major wildlife hospitals in south-east Queensland—The Royal Society for the Prevention of Cruelty to Animals (RSPCA) Queensland Wildlife Hospital, Currumbin Wildlife Hospital, Moggill Koala Rehabilitation Centre and Australia Zoo Wildlife Hospital. The other dataset was from Wildlife Victoria, a state-wide wildlife rescue organisation in Victoria Australia, which had records from 2007 to 2019.

From these two koala datasets, we extracted and examined cases using the key words “livestock”, “cattle/cow/bull/ox”, “horse/stallion/mare”, “sheep”, “goat” and “paddock/farm/grazing land” included in the case description. Data of the koala’s age class (categorised by body mass: Adult (>4 kg), Sub-Adult (2–4 kg) and Young (<2 kg)), sex, location and outcome, admission date and reason, veterinary notes and other field or witness notes were also included if available. We also acknowledge the limitations of data quality presented in these two datasets. The Queensland koala admission database included records of koala age class, sex and outcome that we believe are accurate, but only showed brief comments with limited details regarding the cause of incidents concluded by veterinarians. Therefore, our results relied on veterinarians’ conclusions, which were based on the circumstance and clinical evidence of each case. According to our personal communications with wildlife veterinarians (including A. Gillett, pers. comm., 19 August 2018; C. Lacasse and M. Barrow, pers. comm., 19 August 2021), if a witness report was absent, a conclusion of livestock-related injury/death of koala was only made when supported by both strong clinical evidence and the location of incident, i.e., in a livestock paddock and not on or adjacent to a road, so that the chance of misdiagnoses from vehicle collisions could be minimised. The detailed clinical results and background of incidents were not fully revealed in the KoalaBase.

In contrast to this, the Wildlife Victoria koala rescue record dataset provided limited information about the koala sex, age class and clinical results but had more detailed field observations of livestock–koala incidents. This is because rescue groups acted as the first response, in the field, when incidents were reported by members of the public, and then transported injured animals to hospitals. Therefore, the outcomes of the koalas in the Wildlife Victoria database were shown as dead, sent to hospital (with unknown further information) and unable to be caught.

Following the initial review, all incidents were categorised into two classes: (1) confirmed cases—koalas were witnessed being chased or attacked by livestock in the field, and/or clinical examination results of injured or dead koalas showed convincing evidence of injuries caused by livestock, e.g., abdomen, chest and head traumas [17], when the koala was found in livestock paddocks and not on or adjacent to a road; and (2) possible cases—koalas were found injured, distressed or dead in livestock paddocks without any observation of the cause of the trauma or further clinical information. The outcomes were categorised into Dead—koalas were dead when found, died later or euthanised; Recovered—koalas were treated, rehabilitated then released; and Unknown—koalas were sent to hospital without knowing the final outcome, or were sighted but unable to be caught in the field.

## 3. Results

### 3.1. Online Survey

**Figure 1:**
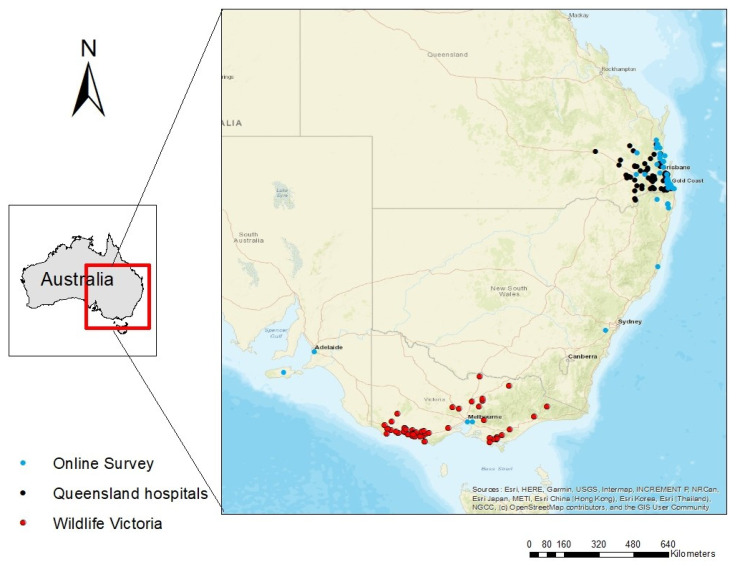
The locations of livestock-related incidents to koalas which were reported by Queensland hospitals, Wildlife Victoria and online survey respondents.

We received 92 completed survey forms, i.e., 11.9% of a total of 775 respondents who started the online survey but did not finish it. This relatively low completion rate may be due to the low occurrence of livestock–koala incidents, with which most survey recipients had little relevant experience. Among the 92 completed respondents, there were 15 veterinarians/nurses, 34 wildlife rescue carers, 20 conservation researchers/staff and 20 farmers. The number of respondents in different categories (had seen confirmed or possible cases, or only heard of anecdotal cases) as well as their evidence, frequency, cattle involvement and koala outcome are shown in Table 1. A map of livestock–koala incidents reported from Queensland wildlife hospitals, Wildlife Victoria and the online survey respondents is shown in Figure 1.

Other than eyewitness reports of the incidents, clinical examination results were another reliable source of evidence to determine if koalas were victims of attacks by livestock. External signs of blunt force trauma included large bruises, bleeding from the mouth, limping and sometimes hoof prints on the koala’s body. Typically, clinical examinations of the injured or dead koala would detect various fractures of the skull, jaw, ribs and pelvis, with abdomen haemorrhage from ruptured internal organs, which were consistent with those reported clinical features of koalas injured by livestock [17].

A total of 28.3% (26/92) of respondents stated that they had handled or seen injured or dead koalas that were definitely attacked by livestock, among which six respondents stated they had witnessed the incident in the field. A further 18 respondents based their testimony on that of the people who had found the koala. A total of 29.3% (27/92) of respondents had handled or seen koalas that were possibly attacked by livestock; the majority (22) were based on the location of koalas, i.e., in livestock paddocks. Another 41.3% (38/92) of respondents had heard of koalas being injured or killed by livestock within their local area; the major sources of this information were wildlife carers (78.9%), hospital/clinic staff (34.2%) and farmers (26.3%). The most common frequency of all livestock–koala incidents (confirmed and possible) was reported as “Only a few cases over the past years”, followed by “1–6 cases every year”. Regarding the outcome of confirmed and possible cases, 45.2% (14/31) of respondents stated that almost none of the koalas involved in incidents survived; 19.4% (6/31) stated that less than half of the koalas survived, whilst 32.3% (10/31) had no knowledge of the koala’s fate. Most (76.9%) respondents with experience of confirmed cases reported that cattle were the type of livestock involved in “Almost all cases” they had seen. Other wildlife, including kangaroos/wallabies, rabbits/hares, snakes, dogs/dingoes, cats, foxes, possums, echidnas, bats, lizards and small marsupial species (e.g., bandicoots) were also reported being killed by livestock.

### 3.2. Admission Records

**Figure 2:**
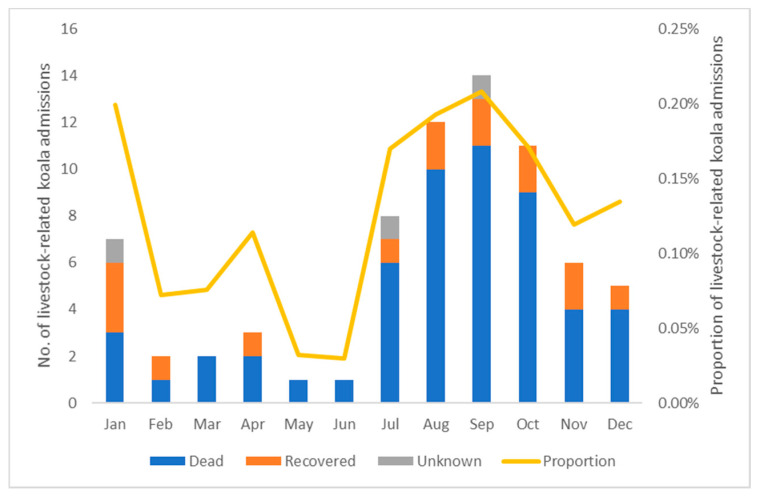
The number of livestock-related koala admissions with their outcomes and proportion of all koala admissions admitted by Queensland wildlife hospitals in each month from 1997 to 2019.

**Figure 3:**
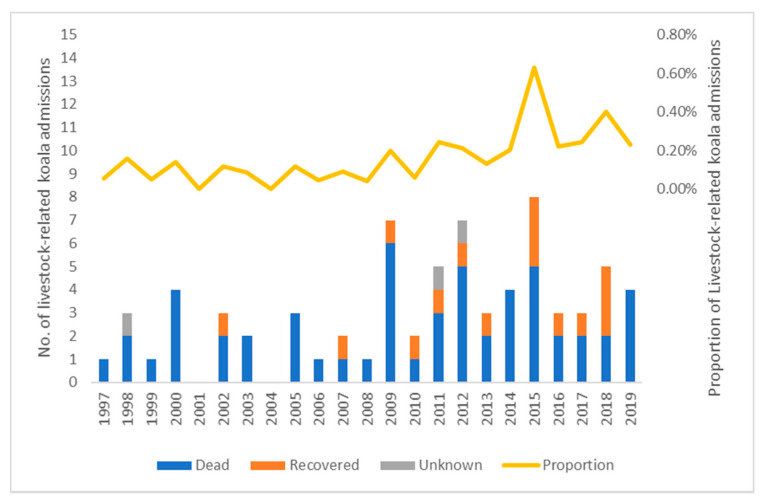
The number of livestock-related koala admissions with their outcomes and proportion of all koala admissions admitted by Queensland wildlife hospitals each year from 1997 to 2019.

In the Queensland Koala Hospital admission database, there were 72 (0.14%) cases of livestock-related incidents out of a total of 50,873 koala admission records from 1997 to 2019 (Table 2). Among those cases, three were sighting records when a koala was reported injured by livestock but unable to be caught. The seasonal trend of livestock-related koala admissions with their outcomes and proportion to total koala admissions throughout the year is shown in Figure 2. The number of livestock–koala incidents (t(8) = −3.878, *p* = 0.004), as well as the proportion to all admissions (X^2^_2_ = 5.484, *p* = 0.064, marginal), increased from the first half of year (January–June) to the second half (July–December). The longitudinal trend of livestock-related koala admissions, with their outcomes and the proportion in each year from 1997 to 2019, is shown in Figure 3. There were on average 3.13 incidents each year: 1.75 before and 4.64 after the start of 2009 (t(17) = −4.129, *p* < 0.001); the proportion of livestock related incidents also had a significant increase after 2009 (X^2^_2_ = 18.788, *p* < 0.001). Of the incidents, 97.2% (70/72) were considered confirmed cases with either field witnesses or clinical evidence (which was, unfortunately, not specified in the database). Only two (2.8%) were considered possible cases as one dead koala was found in a paddock with horses and another koala was found in a paddock with sheep and no further clinical evidence was provided. Of the total confirmed and possible cases, 45.8% (33/72) were females, 29.2% (21/72) were males and 25.0% of the koalas were of unknown sex (18/72), X^2^_1_ = 2.667, *p* = 0.103 (excluding those of unknown sex). In relation to age, 77.8% (56/72) were adults, 12.5% (9/72) were subadults and 9.7% (7/72) were young joeys, X^2^_2_ = 64.083, *p* < 0.001. The outcomes of all the koalas involved were 75% (54/72) dead (including dead when found, died or were euthanised later), 20.8% (15/72) recovered through hospital treatment, and 4.2% (3/72) with unknown fate (e.g., unable to capture in the field), X^2^_1_ = 22.043, *p* < 0.001 (excluding those of unknown fate). Regarding the specific livestock involved in the incidents, cattle accounted for 70.8% (51/72) of all incidents and horses for a further 27.8% (20/72), with a death rate for koalas of 74.5% (38/51) and 75.0% (15/20), respectively. Sheep accounted for one koala death as a possible incident. Based on clinical records, only 26.4% (19/72, X^2^_1_ = 16.056, *p* < 0.001) of koala victims from livestock attack were diagnosed as already diseased in some way, e.g., chlamydiosis, when they were sent to hospitals after the incident.

**Figure 4:**
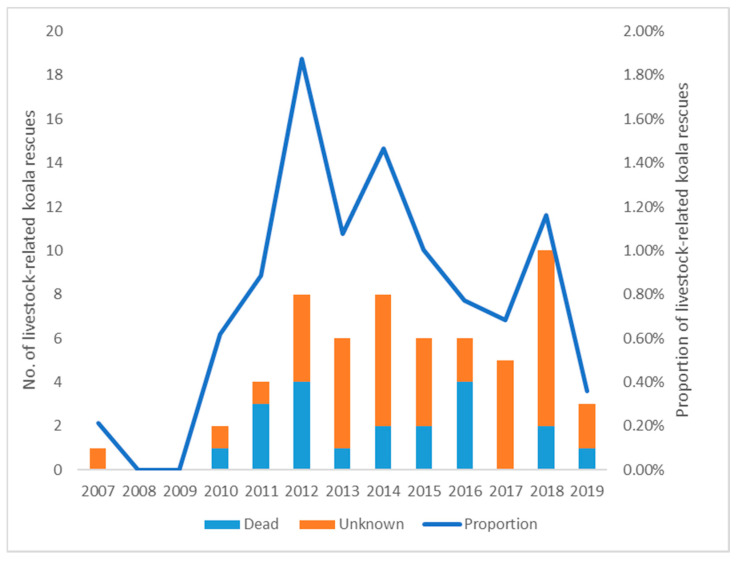
The number of livestock-related koala rescues with their outcomes and proportion of all koalas rescued by Wildlife Victoria each year from 2007 to 2019.

In the Wildlife Victoria koala rescue dataset, there were 59 (0.8%) cases of livestock-related incidents out of a total of 7017 koala rescue records from 2007 to 2019 (Table 3). The number and proportion of annual livestock-related koala rescue records with the outcomes is shown in Figure 4. From 2007 to 2019, there were 4.5 incidents on average each year—0.3 before and 5.8 after the start of 2010 (t(11) = −6.503, *p* < 0.001); the proportion of livestock related incidents also had a significant increase after 2010 (X^2^_2_ = 14.341, *p* < 0.001). Among all incidents, 40.7% (24/59) were confirmed cases with field witnesses, and 59.3% (35/59) were possible cases with no witnesses or clinical information. As an outcome of all the koalas involved, 33.9% (20/59) were dead and 66.1% (39/59) had an unknown fate. Cattle accounted for all the livestock–koala incidents in this dataset. We also found 10 cases where a koala was sighted in a paddock without signs of obvious injuries, and these cases were excluded from the livestock-related incident analyses.

## 4. Discussion

Examination of koala hospital and rescue admission records and responses from the online survey provides convincing evidence of livestock-inflicted injuries and deaths to koalas. In particular, witness statements of first-hand field observations of incidents were given in both admission records and survey responses (eight reports). Details of the incident that were gathered from the online survey and our follow-up emails to those who claimed to be an actual witness, helped provide the incident scenario and describe the general patterns of livestock, mostly cattle, attacks on koalas. Incidents were all witnessed during the day. In general, cattle were reported firstly to be interested in koalas when encountered on the ground and tended to follow and chase koalas if they were moving. Attacks were reportedly mostly initiated by a few leader/dominant animals in the herd without other cattle being involved (according to at least two reports), or could then be joined by other cattle in a form of chase and stampede. The reported observed physical attacks on koalas included being chased, stomped on, head butted with/without horn attacks and the koalas being tossed in the air. Although there are doubts from other survey respondents that cattle only cause harm to koalas by accident due to their bulky body, unrivalled strength and possible ignorance of the small creatures on the ground, these witness descriptions of the incidents have demonstrated that during the time of the incidents, cattle can, and are willing to, initiate actual attacks on koalas. The attack pattern is consistent with bovine aggressive behaviour towards predators [22], indicating that cattle may perceive koalas as potential predators during the attack.

Two veterinarians and koala researchers (A. Gillett and S. Fitzgibbon, pers. comm., 19 August 2018) have both reported that they suspected that such cattle aggression could be triggered by the abnormal walking gaits of koalas on the ground. This was not supported by our survey, as a wide range of wildlife (e.g., kangaroos and possums) were reported to be involved in attacks by livestock. Some veterinarian respondents to the survey suggested that only koalas with predisposing illness are likely to be attacked by livestock, as they are unable to escape due to their weak body condition, and healthy koalas can easily avoid the risk and navigate through farmlands. This suggestion is not supported by koala hospital admission records, as only 26.4% (*p* < 0.001) of livestock-related koala victims were found to be sick, despite no detailed information of possible sub-clinical diseases being provided. From the information provided, we can’t exclude the possibility that koala victims may have had underlying, undetected conditions (e.g., chlamydiosis and retro-virus), which could make them weaker and more vulnerable to livestock attack. However, there were two witness reports from the online survey verifying cattle aggression towards healthy koalas that had just been released by the observers.

Prognoses of koalas with livestock-inflicted injuries were generally poor [17], which was demonstrated in this study by an at least 75% death rate (*p* < 0.001), according to hospital records, and a survival rate reported as “Nearly none” (45.2% of respondents who had experience with such incidents) and “Less than half” (19.4%) in survey responses. Rescue records in Victoria showed a 33.9% koala death rate, which was likely to be underestimated due to the 66.1% unknown outcomes for koalas that were taken to hospital or unable to be caught. Although with lower reported incidence, the death rate for koalas involved in livestock attacks approaches that from vehicle collisions (80.3%) and exceeds that from chlamydiosis (50%) [9,23,24,25].

The average annual frequency of livestock-inflicted incidents to koalas, as determined in this study, was low, i.e., 3.1 cases per year from Queensland hospital records, 4.5 cases per year from Victoria rescue records and “Only a few cases over the past years” by more than 50% of respondents to the survey who have seen or heard of such incidents. The higher frequency of incidents in Victoria is probably due to its greater koala population density compared with that in Queensland [26]. The monthly trend of livestock-related incidents throughout the year in Queensland peaked at the beginning of koala breeding season (August to October). Thus, it was assumed that most victims would be male koalas with an increased activity level in the breeding season, but we found no evidence of such sex bias in this study (*p* = 0.103).

As shown in both Queensland wildlife hospital and Victoria rescue datasets, the annual number of livestock–koala incidents was low before 2009 (Figure 3) or 2010 (Figure 4), but showed an obvious upward trend in the following years where the annual frequency of incidents was at least 2.7 times greater (*p* < 0.001). This coincides with the period when the early media reports about koala mortalities associated with livestock were released [14]. Given there were no significant increases of livestock population in both Queensland and Victoria [27], a possible explanation could be that these media reports facilitated a rise in public awareness about livestock as a potential threat to koalas and resulted in an increased number of livestock-related koala reports. This explanation is supported by the increased proportion of livestock related incidents to the total number in both states (*p* < 0.001). Moreover, it may have been due to greater awareness by koala hospital staff, which could facilitate a drop in the number of misdiagnoses. Lastly, the opening of Australia Wildlife Hospital in 2008 may have contributed to this increase of livestock–koala incidents, with an extra > 500 koalas per year [28] received by hospitals.

Despite the relatively low frequency of reported cases, we believe livestock-related incidents with koalas may be greatly under-reported. For example, evidence was provided from the survey response of a wildlife carer with extensive experience, who stated she had heard of these incidents as early as 2000, from a farmer who witnessed a koala being tossed and trampled by cows, even though the first media report only appeared 10 years later [14]. Factors that could have contributed to this under-reporting of livestock-inflicted incidents with koalas include:

- The Location of Incidents

Livestock attacks on koalas are likely to happen in paddocks, which could be large in scale and generally remote from human activities. Therefore, the likelihood of them being observed, reported and admitted to hospital or rescue groups is extremely low. Reflected in the incident map (Figure 1), the majority of reported incidents were concentrated in human populated areas such as south-east Queensland and southern Victoria. This makes the admission database potentially biased, because other koala victims involved in human activities, e.g., vehicle collision and domestic dog attack, are more likely to be included in the admission database [29]. This bias of admission data is demonstrated by the fact that vehicle collision appeared to be a major reason for hospital admission, according to the records [29], but in a comprehensive study at Koala Coast, where extensive, timely necropsies of koalas were conducted, it is far less than the top two threats to koalas in the wild, i.e., chlamydiosis and wild dog predation [8].

- The Experience of Veterinarians

As livestock attacks on koalas have only emerged in reports anecdotally over the past 10 to 20 years, not all veterinarians have heard of and acknowledge livestock as a source of injury to koalas. This may be because most wildlife hospitals are located along the coastal areas in Queensland, whereas the majority of properties with grazing livestock are concentrated in the western inland regions. Meanwhile, in the absence of field witnesses, injuries caused by livestock can be hard to identify due to their similarities shared with vehicle collision injuries, including fractures of the skull, ribs, chest and long bones, as both are blunt force trauma (except horn attack by cattle) to koalas. Differences between the two cases can be subtle: vehicle collision often causes fractures focused on a region, with shredded claws and superficial abrasions on the limbs and face of a koala when skidding across the road; koalas trampled by livestock may show no external signs of trauma at all but have multiple bone and organ injuries after thorough clinical examinations (A. Hill, V. Nicolson, T. Portas, and M. Barrow, pers. comm., 19 August 2021). Therefore, livestock attacks on koalas have minimal chance of being correctly identified but are more likely to be referred to as vehicle collision or unknown trauma. For example, RSPCA Queensland Wildlife Hospital only added “Livestock Attack” as a reason that could be cited in its wildlife admission record in 2018, and since then six cases have been recorded (up to early 2020).

- Potential Reluctance of Graziers to Report Incidents

Koalas are considered by many Australians as a national icon, and thus their welfare draws much public attention. If an injured or dead koala was found in a paddock, some graziers may be reluctant to report those livestock-inflicted incidents due to the fear of public shaming to their property, while it could also bring negative publicity to the livestock industry. This is at least partly reflected in the survey comments from a few graziers, which denied the possibility of such incidents in an extremely strong and assertive tone, e.g., “To say cattle or livestock injured koalas is a load of bull shit”, and “Who ever made up this story don’t know what they are talking about”. However, other graziers were “truly shocked” by the incident they happened to witness, such as one who reported that they “never would have believed our animals would behave this way if I hadn’t seen it myself”.

Cattle appeared to be the primary threat in livestock-inflicted koala injury incidents, and at least four respondents from the survey who had experience of these incidents suggested that cows in the calving season were most likely to attack koalas. This trend can be seen in Figure 2, i.e., both the number and proportion of livestock-related incidents increased during July–December when most calving occurs in Queensland, which also coincides with increased koala activity due to the beginning of their breeding season. Despite being domesticated animals over many generations, some cattle can be dangerous to humans with their unpredictable temperament, especially during the calving season when cows can be protective of their young [22,30]. In a review of human casualties caused by cattle attack, cows with calves at foot were a key feature associated with the likelihood of cattle attacks on humans [31]. However, cases of koalas being attacked by bulls and cows without calves were also reported by witnesses in the online survey.

Horses, as another grazing livestock, only accounted for 27.8% of the livestock–koala incidents in the Queensland koala hospital admission dataset (Table 2) and were not reported in attacks on koalas in the Victoria Wildlife rescue admission dataset or in the online survey. This is likely due to a much smaller scale of the horse industry (an estimated one million horses [32]) compared with cattle (26.6 million cattle [33]), as well as their less aggressive characteristics [34]. Sheep were only involved in a single possible case from the hospital admission record (Table 2) where a koala was found dead on a sheep station. Other than domestic livestock, wild deer were mentioned in the survey responses to be another possible threat to koalas, but no evidence was provided.

Due to the limited information provided in the database and survey which our study was based on, there were no valid means for us to verify the accuracy and reliability of the data. The reports of confirmed and possible incidents provided substantial evidence, but not solid proof, of the existence of livestock–koala conflict in paddocks. The occurrence and frequency of incidents in Queensland and Victoria only reflected a subset of the real numbers in the two states. The response rate of the survey was relatively low and the actual number of recipients was unknown, but the distribution of completed responses showed a balance among the four recipient groups.

## 5. Conclusions

This study confirms that livestock attack is a koala survival risk worth future attention. The frequency of reported incidents was relatively low but likely to be under-reported. However, it is important to realise that koalas are not necessarily always attacked by livestock when encountered on the ground. Non-agonistic koala–cattle interactions were also reported (by at least two respondents) in the survey, in which cattle approached, sniffed, licked, followed or even chased koalas without actual aggression, consistent with cattle being inquisitive animals [20,22]. Furthermore, koalas are often found to be using eucalypt plantations on farms [35] or inhabiting grazing lands regularly as part of their natural habitat [36]. This implies that koalas and cattle naturally do not, at least not always, see each other as a threat. The increasing urbanisation in Australia may contribute to pushing koalas into contact with livestock in peri-urban areas. Potential factors that may trigger the aggression of cattle towards koalas may include the cattle’s sex, age, breed and for females their calving status. Future research is needed to explore the mechanics and causes behind livestock-inflicted koala injuries and deaths, and to develop management strategies to mitigate livestock as a serious threat to koalas.

## Figures and Tables

**Table 1:** Number of survey respondents who had seen confirmed or possible koala injuries/deaths caused by livestock, and those who had only heard of anecdotal incidents, as well as their evidence, frequency, outcome and cattle involvement. The koala outcome results refer to all livestock-related incidents (Confirmed and Possible cases).

Total Number of Respondents		Confirmed	Possible	Heard
		26	27	38
Evidence	Observed in field	8	n/a	n/a
	Witness report	18	3	n/a
	Location—found in paddocks	n/a	22	n/a
	Clinical evidence	7	7	n/a
Frequency	Only a few cases over the past years	19	16	21
	1–6 cases every year	6	9	12
	7–12 cases every year	0	0	3
	12+ cases every year	1	2	2
Outcome for koala	Almost all survived	0		n/a
	More than half survived	0		n/a
	About half survived	1		n/a
	Less than half survived	6		n/a
	Nearly none survived	14		n/a
	Unknown outcome	10		n/a
Cattle involved	Almost all cases	20	14	n/a
	More than half of the cases	0	1	n/a
	About half of the cases	1	2	n/a
	Less than half of the cases	2	1	n/a
	Nearly none of the cases	1	1	n/a
	Unknown	2	8	n/a

**Table 2:** Summary of confirmed and possible livestock-related incidents to koalas, with the outcomes from each livestock type, and the sex and age class of koalas admitted by Queensland wildlife hospitals from 1997 to 2019. Age class of koalas was categorised by body mass: Adult (>4 kg), Sub-Adult (2–4 kg) and Young (<2 kg). Recovered koalas were those released into the wild after admission and treatment at the hospital.

QLD Koala Hospital	Cattle			Cattle Total	Horses		Horse Total	Sheep	Sheep Total	Grand Total
	Dead	Recovered	Unknown		Dead	Recovered		Dead		
Confirmed	38	10	3	51	14	5	19			70
Adult	29	6	3	38	13	3	16			54
Female	15	1	1	17	6	2	8			25
Male	6	1	1	8	3		3			11
Unknown	8	4	1	13	4	1	5			18
Sub-Adult	5	2		7		2	2			9
Female		2		2		2	2			4
Male	5			5						5
Young	4	2		6	1		1			7
Female	2			2	1		1			3
Male	2	2		4						4
Possible					1		1	1	1	2
Adult					1		1	1	1	2
Female					1		1			1
Male								1	1	1
Grand Total	38	10	3	51	15	5	20	1	1	72

**Table 3:** The summary of confirmed and possible livestock-related incidents to koalas with their outcomes admitted by Wildlife Victoria from 2007 to 2019. The cases with unknown outcome included koalas sent to hospital without information of the final fate, and those observed injured but unable to catch in the field.

Wildlife Victoria	Dead	Unknown	Grand Total
Confirmed	11	13	24
Possible	9	26	35
Grand Total	20	39	59

## Data Availability

The data that support the findings of this study are available upon reasonable request from the corresponding author.

## Appendix A. Survey of Livestock-Inflicted Injuries/Deaths to Koalas

Koala–livestock conflicts are anecdotally reported and koalas with injuries from livestock are admitted to wildlife hospitals throughout the year. This survey aims to investigate the extent and frequency of koalas suffering from livestock-inflicted injuries/deaths in south-east Queensland and other locations in Australia.

Your job:

wildlife veterinarian

wildlife carer

other:

Name of your Organisation/Clinic/Hospital (Optional):

Suburb, Postcode and State:

The estimated time to finish this survey is within 10 min.

1. As part of your job, are you dealing with injured/dead koalas in ways of reporting, handling, caring, rehabilitation or necropsy?
  Yes
  No
2. As an individual (not your hospital/clinic/organisation), how often do YOU receive/process injured/dead koalas every year?
  More than weekly
  Weekly
  Fortnightly
  Monthly
  Less than monthly
  If you have specific details of the number please specify:
3. Have you ever heard of (but NOT been directly involved with or seen) koalas injured or killed by livestock within your local area?
  Yes
  No
4. If yes, how often do you hear of this happening?
  12+ times yearly
  7–12 times yearly
  1–6 times yearly
  only a few times over the past years
5. Where do you hear it from?
  Hospitals or clinics
  Rescue carers
  Farmers
  Social Media
  News (TV, internet, etc.)
  Others (please specify)
6. Have you ever handled (for treatment, rehabilitation or necropsy), or seen, koalas that have DEFINITELY been injured or killed by livestock?
  Yes
  No
7. Of these CONFIRMED cases, how do you know that the koalas were DEFINITELY injured or killed by livestock?
  You have witnessed the incidents when happening
  Testimony from people who found the koala
  Robust clinical evidence
  Others (please specify)
8. What is the robust clinical evidence that confirms the injures/deaths of koalas are caused by livestock? (e.g., livestock hoof prints on koala body, livestock bite marks, etc.)
9. How often do you handle/see such CONFIRMED incidents?
  12+ times yearly
  7–12 times yearly
  1–6 times yearly
  only a few times over the past years.
10. Of these confirmed cases, how many were caused by CATTLE rather than by other livestock?
  Almost all
  More than half
  About half
  Less than half
  Nearly none
  Unable to estimate due to limited evidence
11. Have you ever handled or seen koalas that MAY have been injured or killed by livestock?
  Yes
  No
12. Of these POSSIBLE cases, how do you know these injures/deaths MIGHT have been caused by livestock?
  Case report
  Location of the incident—from livestock paddock
  Clinical evidence, please specify
  Others (please specify)
13. What is the clinical evidence that indicates the injures/deaths of koalas are POSSIBLY caused by livestock?
14. How often do you handle/see such POSSIBLE incidents?
  12+ times yearly
  7–12 times yearly
  1–6 times yearly
  only a few times over the past years.
15. Of these possible cases, how many might have been caused by CATTLE rather than by other livestock?
  Almost all
  More than half
  About half
  Less than half
  Nearly none
  Unable to estimate due to limited evidence
16. Of those koalas possibly or definitely injured by livestock, how many have survived to be released?
  Almost all
  More than half
  About half
  Less than half
  Nearly none
  Don’t know
17. Have you ever seen any other wildlife possibly injured or killed by livestock, and if so please tick the boxes below:
  Never
  Kangaroos/Wallabies
  Rabbits/Hares
  Snakes
  Dogs/Dingoes
  Cats
  Foxes
  Possums
  Echidnas
  Small marsupial species e.g., bandicoots, phascogales, etc.
  Others, please specify
18. If you have seen any cases of koalas injured/killed by livestock please give a brief case description:
19. Any other comments in relation to livestock-inflicted injuries/deaths to koalas, please specify:
20. If you are happy to be contacted for further information, or interested in the final outcome of this survey, please leave your email address below, and a brief report will be sent to you when available.
  Email address

## Appendix B. Livestock–Koala Incident Online Survey Invitation Recipient List

Veterinarians and nurses:

Australia Zoo Wildlife Hospital

Currumbin Wildlife Hospital

Endeavour Veterinary Ecology

Moggill Koala Rehab Centre

RSPCA Queensland

Port Macquarie Koala hospital

Lone Pine Koala Sanctuary

Dreamworld

Taronga Zoo

Taronga Conservation Society Australia

Melbourne Zoo

Zoos Victoria

Adelaide Zoo

Australian Veterinary Board

Australian Veterinary Association

Wildlife Disease Association—Australia

Wildlife Health Australia

Wildlife carers and rescue groups:

WIRES Australian Wildlife Rescue Organisation

Wildlife Preservation Society of Queensland (WPSQ)

Community for Coastal & Cassowary Conservation Inc.

Wildlife Conservancy of Tropical Queensland

Eagles Nest Wildlife Hospital Inc.

Tableland Wildlife Rescue

Tolga Bat Rescue & Research Inc.

Far North Queensland Wildlife Rescue Assoc. Inc.

Magnetic Island Fauna Carers Organisation

Nth Qld Wildlife Care Assoc

Australian Wildlife Rescue Service CQ Inc.

Fauna Rescue Whitsundays Assoc. Inc.

Bush Baby Wildlife Rescue Inc.

Central Queensland Wildlife Hospital Assoc Inc.

Gladstone & District Wildlife Carers Assoc. Inc.

Central Highland Wildlife Carers Inc.

Wildlife Rockhampton- Rescue, Rehabilitation and Release Inc.

Wild on Wildlife Ltd.

Queensland Wildlife Carers & Volunteers Assoc. Inc.

Charleville Animal Rescue & Rehabilitation (CAARER)

Granite Belt Wildlife Carers Inc.

Wildlife Rescue, Rehabilitation & Education Association Inc.

Wildlifes Welfare Carers Inc.

Return to the Wild

Australian Native Animal Rescue and Rehabilitation (ANARRA)

Australian Rescue & Rehabilitation of Wildlife Assoc Inc (ARROW)

Australian Seabird Rescue

Tara Wildlife Support Assoc. Inc.

Wildlife Australian Emergency Rehabilitation & Conservation

Bribie & District Wildlife Rescue Inc.

Wildlife Rehabilitation Centre

Twinnies Pelican and Seabird Rescue Inc.

Wildlife Volunteers Assoc. Sunshine Coast (WILVOS)

Wildlife Rehabilitation Centre

Wildlife Wanderer’s Carer Group Assoc Inc.

Gympie Wildlife Rescue Inc.

Bat Conservation & Rescue Qld. Inc.

Birds Injured Rehabilitated & Orphaned Assoc. Inc. (BIRO)

Brisbane Area Rescue Network Assoc Inc (BARN)

F.A.U.N.A Assoc. Inc.

Ipswich Koala Protection Society Inc.

Orphaned Native Animal Rear & Release Inc (ONARR)

Pelican and Seabird Rescue Inc.

Pine Rivers Koala Care Assoc. Inc.

Qld Native Animal Care

Royal Society For The Prevention Of Cruelty To Animals, Qld Inc.

S.C.R.U.B Catchment Care Group Inc.

South East Qld Wildlife Carers Assoc. Inc.

Wildcare Australia

Paws & Brooks Nature Sanctuary Inc.

Wildlife Preservation Society of Queensland (WPSQ)

Researchers and conservation staff:

Australian National University

Macquarie University

University of New South Wales

University of Sydney

University of Technology, Sydney

Western Sydney University

University of Wollongong

University of Queensland

University of Adelaide

Deakin University

Monash University

University of Melbourne

Department of Agriculture, Water and the Environment, Australian Government

Department of Environment and Science, Queensland Government

Department of Planning, Industry and Environment, New South Wales Government

Department of Environment, Land, Water and Planning, Victoria Government

Department for Environment and Water, South Australia Government

Farm/Livestock/Agriculture Associations:

Cattle Council of Australia

AgForce Queensland

Queensland Agriculture

Landmark Kangaroo Island Livestock

Agriculture Kangaroo Island

Meat & Livestock Australia

Australian Dairy Farmers

Dairy Australia

Beef Central

Queensland Country Life

Queensland Farmers Federation

Victorian Farmers Federation

Australian Farmers
